# Mean distraction force applied in tension‐controlled ligament‐balanced total knee arthroplasty: A systematic review and meta‐analysis

**DOI:** 10.1002/ksa.12629

**Published:** 2025-02-26

**Authors:** Roland Becker, Maximilan Voss, Jonathan Lettner, Robert Hable, Mahmut Enes Kayaalp, Reha Tandogan, Pier Indelli, Nikolai Ramadanov

**Affiliations:** ^1^ Center of Orthopaedics and Traumatology, Brandenburg Medical School University Hospital Brandenburg an der Havel Brandenburg an der Havel Germany; ^2^ Faculty of Health Science Brandenburg Brandenburg Medical School Theodor Fontane Brandenburg an der Havel Germany; ^3^ Faculty of Applied Computer Science Deggendorf Institute of Technology Deggendorf Germany; ^4^ Istanbul Fatih Sultan Mehmet Training and Research Hospital University of Health Sciences Istanbul Turkey; ^5^ Ortoklinik & Çankaya Hospital Ankara Turkey; ^6^ Department Orthopaedic Surgery Südtiroler Sanitätsbetrieb Brixen Italy; ^7^ Institute of Biomechanics Paracelsus Medical University (PMU) Salzburg Austria

**Keywords:** distraction force, ligament tension, replacement, total knee arthroplasty

## Abstract

**Purpose:**

Proper tension of the collateral ligaments is the key to success in total knee arthroplasty (TKA). The study aimed to identify the distraction force for the medial and lateral femorotibial compartments in tension‐controlled ligament‐balanced TKA at 0° and 90° of knee flexion.

**Methods:**

A literature search was conducted in PubMed up to 31 December 2024 to identify studies that reported exact values of the distraction force applied in tension‐controlled ligament‐balanced TKA. Mean distraction force at 0° and 90° of knee flexion were calculated for the native knee, cadaver knee, and computer model/artificial knee groups. Differences between groups were calculated using Kruskal–Wallis and Mann–Whitney *U* tests, with *p* ≤ 0.05 considered significant. A frequentist meta‐analysis of subgroup analysis between native and cadaver knee studies was performed using a random effects model with inverse variance and the Sidik–Jonkman heterogeneity estimator with Hartung–Knapp adjustment to calculate participant age and sex.

**Results:**

Out of 116 included primary studies involved, a total of 6869 participants had distraction force measurements during TKA. The mean distraction force was 149.9 N (35.0–320.0 N) at 0° knee extension and 139.5 N (14.7–244.7 N) at 90° of flexion. Using the Kruskal–Wallis test or the Mann–Whitney *U* test, there were no significant differences in distraction force between native knee, cadaver knee, computer model/artificial knee studies at extension (*p* = 0.2480 and *p* = 0.1130) and at 90° of knee flexion (*p* = 0.8439 and *p* = 0.6241).

**Conclusion:**

This meta‐analysis is the first to quantify distraction force in TKA, providing essential reference values of 149.9 N at 0° extension and 139.5 N at 90° flexion. These findings offer valuable guidelines for intraoperative soft tissue management during TKA procedures. The consistency of distraction force across different experimental models suggests that these values are broadly applicable. However, it remains unclear whether a more personalized distraction force should be considered for gap preparation.

**Level of Evidence:**

Level IV.

**Abbreviations**
BMI: body mass index
CI: confidence interval
PRISMA: Preferred Reporting Items for Systematic Reviews and Meta‐Analyses
PROSPERO: International Prospective Register of Systematic Reviews
RCT: randomized controlled trial
RoB: risk of bias
ROBINS‐I: Risk Of Bias In Non‐randomized Studies of Interventions
TKA: total knee arthroplasty

## INTRODUCTION

The kinematics of the knee depends on both the bony contour such as the shape of the distal femur and the proximal tibia but also the soft tissue. Mediolateral and rotational stability are provided by the ligaments and capsule. Apart from measured bone resection, gap balancing is the key in total knee arthroplasty (TKA) to achieve mediolateral balance and good function [132]. The determination of mean distraction force at extension and 90° is of considerable clinical importance since stiffness and instability are major causes for revision TKA. A prospective study of 18,065 knees revealed the need for revision of 405 knees. The reason for failure was infection (25.7%), instability (24.4%), aseptic loosening (21.2%), stiffness (14.1%) and multiple other reasons [110].

To ensure adequate soft tissue tension in TKA, the gap balancing technique was introduced in the 90s using a spacer or ligament tensor inserted intraoperatively in the medial and lateral femorotibial compartment in both extension and flexion to guarantee rectangular gaps. Some of the tensor devices are more sophisticated and allow measurement of femorotibial distraction under the defined force applied separately to the medial and lateral compartments in both extensions and 90° of flexion. However, the amount of distraction force applied during surgery is still unclear depending rather on surgical experience and subjective perception. Currently, the definition of soft tissue balance of the knee is poorly defined with little consensus available in the literature.

In order to help clarify data of the literature synthesized comparing the intraoperative data with those obtained from human cadavers, artificial specimens, or computer models.

A systematic review and meta‐analysis were conducted attempting to establish accurate values by synthesizing as much primary data as possible.

The aim of the study was to determine the mean distraction force values for the medial and lateral compartments in tension‐controlled ligament‐balanced TKA at both extension and 90° of flexion.

## METHODS

### Reporting guidelines and protocol registration

The study protocol has been registered in the International Prospective Register of Systematic Reviews (PROSPERO) with the registration number CRD42024578708. We strictly followed the updated PRISMA guidelines [107] and meta‐analysis guidelines [112].

### Data sources and search strategies

A literature search was conducted up to 31 December 2024 in PubMed, Embase and Epistemonikos. A Boolean search strategy was used to identify studies reporting exact values of the distraction force applied in tension‐controlled ligament‐balanced TKA. The search strategy was specifically tailored to the syntax of each database: (((total knee arthroplasty) OR (TKA) OR (knee replacement)) AND ((balance) OR (balanced) OR (balancing) OR (distraction))). There were no restrictions on the year of publication or language.

### Study screening and selection

In a stepwise screening and selection process, three independent reviewers (MV, JL and NR) assessed the titles and abstracts of the identified records for eligibility. Studies were included in the final meta‐analysis after full‐text analysis by these reviewers. Disagreements were resolved by discussion and consensus with a fourth reviewer (RB). The kappa coefficient (*κ*) was calculated to assess inter‐reviewer agreement.

### Inclusion and exclusion criteria

The following inclusion criteria were applied: (1) study design: primary studies including randomized controlled trials (RCTs), observational studies, case series and case reports; (2) participants: human adults, human cadavers, artificial specimens and computer models; (3) intervention: TKA; (4) outcome parameters: distraction force applied to the medial and lateral compartment of the knee by the ligament tensor at full leg extension or 90° leg flexion.

The following exclusion criteria were applied: (1) primary studies that did not report the outcome of interest; (2) all types of reviews and editorials; (3) studies that reported incomplete data to an extent that compromised the credibility of the results.

### Data extraction and analysis

Data extraction was performed by two independent reviewers (NR and MV) and proofed by a third reviewer (RB). Disagreements were resolved by discussion and consensus with the third reviewer (RB). The following data were extracted: first author, year of publication, study origin, number of participants included, number of knees operated, use of navigation systems, indication for surgery, participant characteristics, study design, information to perform risk of bias assessment and exact values of distraction force applied to the medial and lateral compartment of the knee by the ligament tensor at full leg extension or 90° of flexion. In the case of missing standard deviations (SDs), the SD value was added using the rule of thumb SD = range/4 if the range was given. Otherwise, the missing standard deviation was added by imputation. These data were then exported from the included primary studies into an Excel spreadsheet for appropriate statistical analysis.

### Outcome: Distraction force

To collect more consistent data, full leg extension was defined as knee flexion <20°. If the primary studies provided multiple values, we extracted the value that was 0° or closest to 0°. Similarly, 90° of knee flexion was defined within a range of 60–120° of knee motion. If the primary studies provided multiple values, we extracted the value that was 90° or closest to 90°. Only one value was extracted per full extension and 90°flexion. In cases where the distraction force was reported separately for the medial and lateral knee compartments, both values were summed, and the summed value was extracted. In cases where the distraction force was reported in a range of applied values (e.g., 100–200 N), a quasi‐linear correlation was assumed, and a mean value of 150 N was chosen. In cases where the distraction force was reported in pounds (lb) or kilograms (kg), these units were converted to Newtons (N). In cases where no range of motion information was provided, the extracted distraction force value was considered to be measured at extension. The summed, averaged and converted values are highlighted in Table 1.

**Table 1:** Details on study and participant characteristics.

Study	Year of publication	Origin of publication	Study type	Specimen	Robot assistance or navigation application	Participants, *N*	Knees, *N*	Surgical indication (as % of number of participants)			Participant age (in years ± SD; range)	Male sex as % of number of participants	BMI (in kg/m² ± SD; range)	Distraction load in N at full extension	Distraction load in N at 90° of flexion	Special features of distraction load data extraction
								Osteoarthritis (%)	Rheumatoid arthritis (%)	Avascular necrosis or other indications (%)						
Amiri et al. [1]	2012	Canada	Experimental	Computer model/artificial knee	No	1	1	NR	NR	NR	NR	NR	NR	100	100	‐
Asano et al. [2]	2004	Japan	Retrospective case series	Native knee	No	77	77	72.7	27.3	‐	68.9; 44–85	15.6	NR	127	121	‐
Babazadeh et al. [3]	2014	Australia	RCT	Native knee	Yes	51	51	88.2	9.8	2.0	69.9 ± 8.5; NR	27.5	31.1 ± 5.1; NR	118	NR	1
Bardou‐Jacquet et al. [4]	2021	France	Prospective case series	Native knee	Yes	29	29	100	‐	‐	70.0 ± 9.5; 45–89	48.3	28.0 ± 3.5; 22–34	89	NR	2; 3
Barink et al. [5]	2004	Netherlands	Experimental	Computer model/artificial knee	No	1	1	NR	NR	NR	NR	NR	NR	100	100	‐
Basselot et al. [6]	2016	France	Experimental	Cadaveric knee	No	13	13	NR	NR	NR	NR	NR	NR	150	NR	‐
Bäthis et al. [7]	2004	Germany	Prospective case series	Native knee	Yes	22	22	NR	NR	NR	63.8; 49–82	36.4	NR	100	NR	‐
Burkhart et al. [10]	2017	Canada	Experimental	Cadaveric knee	No	6	12	NR	NR	NR	76.5 ± 11.6; NR	NR	NR	100	100	2
Christen et al. [11]	2007	Switzerland	Prospective case series	Native knee	No	83	91	NR	NR	NR	71.0 ± 9.1; NR	27.7	NR	200	200	2
Christen et al. [12]	2017	Switzerland	Prospective comparative study	Native knee	No	87	88	100	‐	‐	67.0; NR	31.0	NR	200	200	2
Ferreira et al. [17]	2017	Brazil	Experimental	Cadaveric knee	No	22	22	NR	NR	NR	NR	NR	NR	100	80	‐
Fitz et al. [18]	2012	USA	Therapeutic study	Native knee	No	10	10	NR	NR	NR	65.6; 60–83	20.0	NR	NR	90	‐
Foge et al. [19]	2019	USA	Experimental	Cadaveric knee	Yes	5	10	NR	NR	NR	NR	NR	NR	178	178	1
Fujimoto et al. [20]	2012	Japan	Retrospective cohort study	Native knee	No	90	100	NR	NR	NR	76.2 ± 6.1; 58–93	15.6	26.4 ± 3.4; NR	178	178	1
Gejo et al. [21]	2010	Japan	Experimental	Cadaveric knee	No	NR	8	NR	NR	NR	NR; 65–85	NR	NR	100	100	‐
Gejo et al. [22]	2008	Japan	Therapeutic study	Native knee	No	17	20	NR	NR	NR	68.0; 44–84	11.8	NR	178	178	1
Grifka et al. [23]	2020	Germany	Experimental	Cadaveric knee	Yes	NR	10	NR	NR	NR	78.4 ± 5.5; NR	NR	NR	90	90	‐
Hananouchi et al. [25]	2011	Japan	Prospective case series	Native knee	No	73	73	NR	NR	NR	74.5; 58–88	16.4	NR	133	133	1
Heesterbeek et al. [26]	2010	Netherlands	Prospective case series	Native knee	Yes	50	50	NR	NR	NR	63.0; 44–84	36.0	NR	150	NR	2
Heesterbeek et al. [27]	2009	Netherlands	Prospective cohort study	Native knee	No	83	87	NR	NR	NR	65.8 ± 9.2; NR	22.9	NR	200	150	‐
Heesterbeek et al. [28]	2011	Netherlands	Prospective cohort study	Native knee	Yes	49	49	100	‐	‐	62.0 ± 8.8; NR	38.8	NR	150	100	‐
Heesterbeek et al. [30]	2010	Netherlands	Retrospective cohort study	Native knee	Yes	54	54	NR	NR	NR	NR	35.2	NR	150	100	‐
Heesterbeek et al. [31]	2015	Netherlands	Prospective cohort study	Native knee	Yes	80	80	100	‐	‐	71.0 ± 9.7; NR	31.3	NR	160	140	‐
Higuchi et al. [33]	2009	Japan	RCT	Native knee	No	68	76	100	‐	‐	68.4; 56–81	27.9	NR	80	80	‐
Inui et al. [34]	2018	Japan	Retrospective case series	Native knee	Yes	62	62	100	‐	‐	72.5 ± 9.3; NR	12.9	NR	160	160	3
Ishida et al. [35]	2018	Japan	Retrospective cohort study	Native knee	Yes	57	57	100	‐	‐	78.0; 63–92	15.8	NR	178	178	1
Itokazu et al. [37]	2016	Japan	Retrospective case series	Native knee	No	47	48	100	‐	‐	74.0 ± 6.0; NR	6.4	NR	120	120	‐
Itou et al. [38]	2023	Japan	Retrospective case series	Native knee	No	50	55	78.0	12.0	10.0	72.9 ± 6.7; NR	32.0	26.6 ± 4.2; NR	NR	15	1
Jawhar et al. [39]	2016	Germany	Retrospective case series	Native knee	Yes	81	81	100	‐	‐	70.0 ± 6.0; NR	NR	NR	150	150	‐
Jeffcote et al. [40]	2007	Australia	Experimental	Cadaveric knee	No	5	5	NR	NR	NR	NR	NR	NR	100	100	‐
Jia et al. [41]	2023	China	Retrospective cohort study	Native knee	No	92	92	100	‐	‐	65.7 ± 5.3; NR	21.7	27.5 ± 3.2; NR	100	NR	‐
Johnston et al. [42]	2019	United Kingdom	Experimental	Computer model/artificial knee	No	N/A	3	NR	NR	NR	NR	NR	NR	154	NR	2
Joseph et al. [43]	2013	Australia	RCT	Native knee	Yes	40	41	NR	NR	NR	66.8; 52–86	40.0	NR	135	135	‐
Kamei et al. [45]	2011	Japan	Prospective case series	Native knee	No	23	24	95.8	4.2	‐	77.0; 68–84	21.7	NR	133	NR	1
Keshmiri et al. [49]	2015	Germany	Retrospective case series	Native knee	No	40	40	100	‐	‐	65.0; 47–89	52.5	NR	90	90	‐
Kim et al. [50]	2017	South Korea	RCT	Native knee	No	98	98	100	‐	‐	68.6 ± 5.9; 67–70	4.1	NR	111	NR	1; 2
Koh et al. [51]	2020	South Korea/USA	Experimental	Cadaveric knee	No	7	14	NR	NR	NR	65.5 ± 4.0; 58–70	71.4	NR	200	200	‐
Koh et al. [52]	2019	South Korea	Experimental	Computer model/artificial knee	No	N/A	1	NR	NR	NR	NR	NR	NR	133	133	‐
Kuster et al. [53]	2009	Australia/Switzerland	Experimental	Cadaveric knee	No	NR	6	NR	NR	NR	NR	NR	NR	100	NR	‐
Kwak et al. [54]	2012	South Korea	Experimental	Cadaveric knee	No	NR	5	NR	NR	NR	NR	NR	NR	150	NR	‐
Lee et al. [55]	2010	South Korea	RCT	Native knee	Yes	116	116	100	‐	‐	66.5; 50–81	4.3	NR	178	178	1
Lee et al. [57]	2017	South Korea	RCT	Native knee	No	50	50	100	‐	‐	70.8; 56–86	2.0	25.6; 20.7–31.2	89	NR	1
Ma et al. [59]	2017	Japan	Retrospective case series	Native knee	No	50	50	100	‐	‐	NR	20.0	NR	89	89	1
Malavolta et al. [61]	2018	Italy	Prospective case series	Native knee	No	87	87	NR	NR	NR	NR	33.3	29.0; NR	320	NR	‐
Marmignon et al. [63]	2005	France	Experimental	Cadaveric knee	Yes	NR	2	NR	NR	NR	NR	NR	NR	200	NR	2; 3
Matsui et al. [65]	2021	Japan	Prospective cohort study	Native knee	No	94	104	87.5	3.8	8.7	77.9 ± 5.0; 53–89	12.8	25.2; 15.6–36.4	130	130	‐
Matsui et al. [66]	2016	Japan	Experimental	Cadaveric knee	No	3	6	NR	NR	NR	NR	0.0	NR	130	NR	‐
Matsumoto et al. [67]	2013	Japan	Therapeutic study	Native knee	Yes	120	120	100	‐	‐	74.3 ± 1.0; NR	8.33	NR	178	178	1
Matsumoto et al. [68]	2014	Japan	Retrospective case series	Native knee	Yes	120	135	100	‐	‐	74.6; 58–88	11.7	NR	222	NR	1; 2
Matsumoto et al. [69]	2011	Japan	RCT	Native knee	No	40	40	100	‐	‐	73.7 ± 1.5; NR	0.0	NR	178	178	1
Matsumoto et al. [70]	2009	Japan	Retrospective case series	Native knee	Yes	30	30	100	‐	‐	73.3 ± 7.4; NR	0.0	NR	245	245	1; 2
Matsumoto et al. [71]	2023	Japan	Retrospective cohort study	Native knee	No	70	70	100	‐	‐	74.7; 52–88	15.7	25.9 ± 4.0; NR	178	178	1
Matsumoto et al. [72]	2023	Japan	Retrospective cohort study	Native knee	Yes	60	60	100	‐	‐	72.7 ± 8.1; NR	16.7	26.8 ± 3.8; NR	178	178	1
Matsumoto et al. [73]	2016	Japan	Retrospective case series	Native knee	Yes	30	30	100	‐	‐	79.1; 66–87	10.0	NR	150	150	‐
Matsumoto et al. [74]	2017	Japan	Retrospective case series	Native knee	Yes	35	35	100	‐	‐	75.5 ± 5.4; 61–83	17.1	NR	178	178	1
Matsuzaki et al. [75]	2013	Japan	Retrospective case series	Native knee	Yes	30	30	100	‐	‐	73.9 ± 6.3; 61–89	NR	NR	178	178	1
Matziolis et al. [77]	2016	Germany	RCT	Native knee	Yes	56	60	100	‐	‐	66.7 ± 9.0; 49–87	41.1	NR	200	NR	‐
Matziolis et al. [78]	2024	Germany	Experimental	Cadaveric knee	No	NR	1	NR	NR	NR	NR	NR	NR	140	160	‐
Meere et al. [79]	2016	USA	Retrospective case series	Native knee	Yes	101	101	NR	NR	NR	NR	NR	NR	133	133	2
Minoda et al. [80]	2012	Japan	Prospective case series	Native knee	No	80	100	100	‐	‐	75.1; 60–88	12.5	25.9; NR	120	NR	‐
Minoda et al. [81]	2015	Japan	Retrospective case series	Native knee	No	259	259	100	‐	‐	76.3; 47–88	17.8	26.2; NR	120	120	‐
Morishige et al. [83]	2009	USA	Experimental	Cadaveric knee	Yes	5	10	NR	NR	NR	70.6; 55–81	80.0	NR	120	NR	‐
Moro‐oka et al. [84]	2010	Japan	Retrospective case series	Native knee	No	17	14	NR	NR	NR	NR	NR	NR	256	NR	‐
Muratsu et al. [85]	2010	Japan	Retrospective case series	Native knee	No	80	80	100	‐	‐	74.5 ± 6.2; NR	21.3	NR	245	NR	1; 2
Nagai et al. [86]	2018	Japan	Retrospective case series	Native knee	No	275	275	100	‐	‐	74.9; NR	16.7	25.7; NR	178	178	1
Nakamura et al. [90]	2018	Japan	Retrospective case series	Native knee	No	50	50	100	‐	‐	77.6 ± 5.4; NR	10.0	26.2 ± 3.8; NR	NR	178	‐
Nakano et al. [91]	2016	Japan	Retrospective case series	Native knee	Yes	55	55	100	‐	‐	74.2 ± 7.3; NR	21.8	NR	178	178	1
Nicholls et al. [93]	2007	Australia/Switzerland	Experimental	Cadaveric knee	No	NR	6	NR	NR	NR	NR	NR	NR	100	100	‐
Niki et al. [94]	2015	Japan	Retrospective case series	Native knee	No	36	39	89.7	10.3	‐	69.8 ± 14.5; NR	25.0	25.3 ± 3.1; 21.6–30.9	178	178	1
Nishizawa et al. [95]	2013	Japan	RCT	Native knee	No	40	40	NR	NR	NR	73.6 ± 5.8; NR	75.0	NR	178	NR	1
Nowakowski et al. [96]	2011	Switzerland	Experimental	Cadaveric knee	No	5	10	NR	NR	NR	29.5 ± 7.6; NR	100	NR	175	175	2
Ogawa et al. [98]	2022	Japan	Retrospective case series	Native knee	Yes	43	43	100	‐	‐	70.7 ± 6.4; NR	44.2	25.5 ± 3.4; NR	120	120	‐
Okamoto et al. [100]	2013	Japan	Retrospective cohort study	Native knee	No	70	90	100	‐	‐	75.0; 51–89	35.7	NR	176	NR	‐
Okamoto et al. [101]	2014	Japan	Prospective comparative study	Native knee	No	59	75	100	‐	‐	77.0; 51–89	10.2	NR	176	NR	‐
Okamoto et al. [102]	2016	Japan	Retrospective comparative study	Native knee	No	48	54	100	‐	‐	73.8; 59–83	8.3	NR	196	NR	1
Orsi et al. [104]	2022	USA/Australia	Retrospective cohort study	Native knee	Yes	141	141	100	‐	‐	67.5 ± 8.7; 50–94	61.0	31.8 ± 5.8; 21.0–48.4	80	80	‐
Oshima et al. [105]	2021	Japan	Retrospective case series	Native knee	No	30	30	100	‐	‐	73.0 ± 9.6; 50–85	33.3	27.0 ± 4.9; 21.7–33.3	160	140	‐
Oshima et al. [106]	2020	Japan	Retrospective cohort study	Native knee	No	41	41	100	‐	‐	76.0 ± 6.6; 60–88	24.4	25.6 ± 3.4; 18.6–32.2	120	120	3
Roth et al. [116]	2015	USA	Experimental	Cadaveric knee	No	NR	10	NR	NR	NR	NR	NR	NR	100	100	‐
Sasaki et al. [118]	2012	Japan	Retrospective case series	Native knee	No	30	30	100	‐	‐	74.6 ± 6.5; NR	13.3	NR	178	178	1
Schirm et al. [119]	2010	Switzerland/Australia	Experimental	Cadaveric knee	No	NR	14	NR	NR	NR	NR	NR	NR	100	NR	‐
Seito et al. [120]	2021	Japan	Retrospective case series	Native knee	Yes	55	55	100	‐	‐	73.5 ± 6.8; NR	29.1	27.3 ± 4.4; NR	111	111	1; 2
Seo et al. [121]	2017	South Korea	Retrospective case series	Native knee	No	40	40	100	‐	‐	70.3; 52–89	NR	25.2 ± 2.4; 20.5–32.9	133	NR	1
Seon et al. [122]	2011	South Korea	Prospective cohort study	Native knee	No	88	112	100	‐	‐	67.6; 52–79	10.2	NR	200	200	‐
Shalhoub et al. [123]	2019	USA	Retrospective case series	Native knee	Yes	117	121	NR	NR	NR	68.0 ± 6.0; NR	44.4	29.0 ± 5.0; NR	85	85	2; 3
Shalhoub et al. [124]	2018	USA	Experimental	Cadaveric knee	Yes	14	14	NR	NR	NR	73.0 ± 13.0; NR	71.4	26.8 ± 7.6; NR	180	130	2; 3
Shin et al. [125]	2020	South Korea	Prospective case series	Native knee	Yes	114	122	100	‐	‐	71.5 ± 6.5; NR	10.5	NR	35	35	‐
Song et al. [126]	2021	South Korea	Prospective cohort study	Native knee	No	90	90	100	‐	‐	NR	8.9	NR	200	NR	1; 2
Sriphirom et al. [127]	2017	Thailand	Prospective cohort study	Native knee	Yes	40	40	100	‐	‐	NR	NR	NR	196	NR	1
Takagi et al. [132]	2020	Japan	Retrospective case series	Native knee	Yes	35	35	100	‐	‐	74.3 ± 7.0; NR	14.3	NR	133	133	1
Takahashi et al. [133]	2022	Japan	Retrospective cohort study	Native knee	Yes	73	73	100	‐	‐	72.5 ± 7.4; 43–87	35.6	NR	132	132	‐
Takashima et al. [134]	2020	Japan	Retrospective case series	Native knee	Yes	48	51	100	‐	‐	74.7 ± 1.3; NR	18.8	25.3 ± 1.7; NR	178	NR	1
Tanaka et al. [137]	2007	Japan	Experimental	Cadaveric knee	No	10	10	NR	NR	NR	NR; 72–92	60.0	NR	178	178	1
Tang et al. [138]	2017	China	Prospective cohort study	Native knee	No	45	50	100	‐	‐	64.0 ± 7.0; NR	28.9	26.5 ± 4.4; NR	150	150	‐
Terashima et al. [140]	2014	Japan	Retrospective cohort study	Native knee	Yes	30	30	100	‐	‐	71.9 ± 7.7; 55–85	20.0	24.5 ± 1.8; 18.9–34.9	160	NR	3
Tsuboska et al. [141]	2019	Japan	Prospective case series	Native knee	No	65	130	100	‐	‐	75.7; 65–88	15.4	NR	178	178	1
Tsuboska et al. [142]	2017	Japan	Retrospective cohort study	Native knee	No	123	140	100	‐	‐	74.0; 60–87	11.4	NR	178	NR	1
Tsukada et al. [143]	2012	Japan	Retrospective case series	Native knee	No	504	504	88.3	9.9	1.8	71.7 ± 7.4; NR	16.5	26.3 ± 3.9; NR	120	120	2
Tsukada et al. [144]	2019	Japan	Retrospective case series	Native knee	No	107	107	97.2	2.8	‐	75.0 ± 8.0; NR	22.4	26.0 ± 4.0; NR	133	133	1
Viskontas et al. [146]	2007	Canada	Experimental	Cadaveric knee	Yes	8	8	NR	NR	NR	77.0; NR	62.5	NR	NR	100	‐
Völlner et al. [147]	2019	Germany	Experimental	Cadaveric knee	Yes	NR	10	NR	NR	NR	NR	NR	NR	180	180	‐
Wada et al. [148]	2019	Japan	Experimental	Cadaveric knee	Yes	6	6	NR	NR	NR	82.0; 61–91	83.3	NR	NR	178	2
Wakama et al. [149]	2019	Japan	Retrospective case series	Native knee	No	50	59	100	‐	‐	74.0; 69–78	NR	25.0; 23.4–29.3	156	NR	1; 2
Wakelin et al. [151]	2023	USA	Prospective cohort study	Native knee	Yes	310	310	100	‐	‐	68.0 ± 8.0; NR	41.6	31.0 ± 5.0; NR	80	80	2
Walde et al. [152]	2010	Germany	Prospective case series	Native knee	Yes	93	93	100	‐	‐	67.1; 31–84	41.9	NR	90	NR	‐
Walker et al. [153]	2014	USA	Experimental	Cadaveric knee	Yes	NR	10	NR	NR	NR	NR	NR	NR	145	NR	‐
Watanabe et al. [154]	2015	Japan	Retrospective case series	Native knee	Yes	34	44	81.8	18.2	NR	71.0 ± 7.0; NR	23.5	NR	178	178	1
Fan et al. [155]	2023	USA	Retrospective case series	Native knee	Yes	273	273	NR	NR	NR	NR	NR	NR	180	NR	‐
Wyss et al. [157]	2008	Switzerland	Retrospective case series	Native knee	No	95	109	NR	NR	NR	72.9 ± 7.4; 55–92	27.4	NR	225	NR	2
Yamagami et al. [159]	2021	Japan	Retrospective case series	Native knee	Yes	113	113	97.3	0.9	1.8	71.2 ± 9,4; 31–87	15.9	26.8 ± 4.1; 17.7–37.0	140	140	2
Yoon et al. [161]	2013	South Korea	Prospective case series	Native knee	Yes	122	128	100	‐	‐	68.0 ± 2.6; NR	NR	NR	178	178	‐
Yoon et al. [162]	2014	South Korea	Prospective cohort study	Native knee	Yes	50	50	100	‐	‐	68.0 ± 2.1; NR	8.0	NR	150	150	‐
Zapata et al. [164]	2019	USA/Belgium	Experimental	Computer model/artificial knee	No	N/A	1	NR	NR	NR	NR	NR	NR	230	230	3
Zhao et al. [165]	2021	China	Prospective cohort study	Native knee	No	48	60	100	‐	‐	66.0; 56–76	NR	NR	89	89	1
Zhao et al. [166]	2023	China	Retrospective case series	Native knee	No	60	73	NR	NR	NR	67.5 ± 5.9; 57–82	13.3	NR	NR	100	‐
Zimmermann et al. [168]	2012	Germany	Experimental	Cadaveric knee	Yes	NR	1	NR	NR	NR	NR	NR	NR	100	NR	2; 3

*Note*: 1: Conversion from other units to N; 2: Calculation of the mean value from a reported range; 3: Addition of reported values.

Abbreviations: BMI, body mass index; N, Newtons; N/A, not applicable; NR, not reported; RCT, randomized controlled trial; SD, standard deviation.

### Quality assessment

Two independent reviewers (JL and MV) individually assessed the quality of the primary studies included in the meta‐analysis. The risk of bias (RoB) was assessed using the Cochrane RoB 2 tool [129] for RCTs and the Risk of Bias In Non‐Randomized Studies of Interventions (ROBINS‐I) tool [128] for non‐randomized studies. Disagreements between the reviewers were resolved by discussion and consensus with a third reviewer (NR, RB). Publication bias was calculated using the Begg and Egger test [32]. In addition, publication bias is presented in funnel plots.

### Statistics

Mean distraction force at extension and 90° of knee flexion was calculated for the native knee, cadaver knee and computer model/artificial knee. As the distraction force data were not normally distributed and the number of studies per group (native knee, cadaver knee, computer model or artificial knee) was very different, the Kruskal–Wallis test was calculated as a non‐parametric alternative to ANOVA. Due to the small number of computer model or artificial knee studies, a separate comparison only between native and cadaver knee studies was calculated using the Mann–Whitney *U* test as a non‐parametric alternative to the *t* test, with *p* < 0.05 considered significant.

A frequentist meta‐analysis of subgroup analysis between native and cadaver knee studies was performed using a random effects model with inverse variance and the Sidik‐Jonkman heterogeneity estimator with Hartung–Knapp adjustment to calculate participant age and sex. It was not possible to output a forest plot as the number of primary studies was too large for a meaningful visualization. To assess the impact of primary studies with a high risk of bias included in the meta‐analysis, a sensitivity analysis was conducted by excluding studies identified as having a high risk of bias. All statistical calculations were performed by an experienced statistician (RH). These analyses were performed using the R packages meta and metafor.

## RESULTS

### Study identification and selection

**Figure 1:**
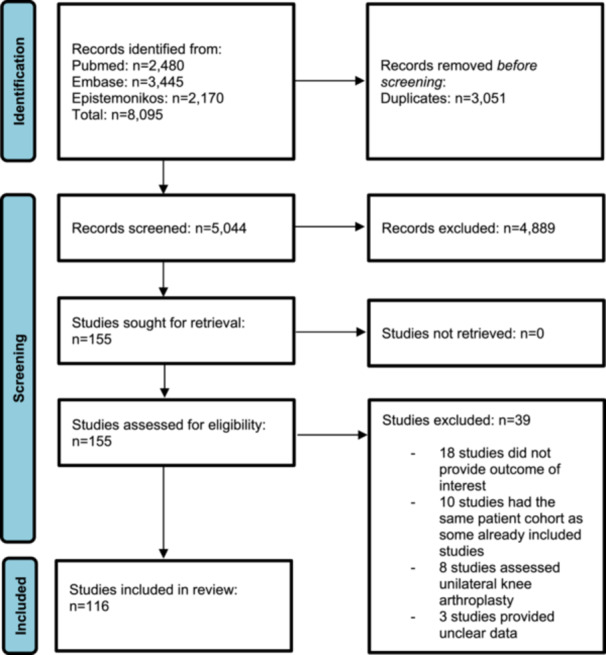
PRISMA flow chart diagram. PRISMA, Preferred Reporting Items for Systematic Reviews and Meta‐Analyses.

A description of the study selection process is given in a PRISMA flowchart (Figure 1). The search of PubMed, Embase and Epistemonikos up to 31 December 2024 yielded 8095 records. In an initial screening by title and abstract, 155 were assessed for eligibility with high inter‐rater agreement (*κ* = 0.92). Of these, 39 were excluded after the second screening by full‐text analysis (*κ* = 1.0), leaving a total of 116 studies on distraction force in TKA [1, 2, 3, 4, 5, 6, 7, 10, 11, 12, 17, 18, 19, 20, 21, 22, 23, 25, 26, 27, 28, 30, 31, 33, 34, 35, 37, 38, 39, 40, 41, 42, 43, 45, 49, 50, 51, 52, 53, 54, 55, 57, 59, 61, 63, 65, 66, 67, 68, 69, 70, 71, 72, 73, 74, 75, 77, 78, 79, 80, 81, 83, 84, 85, 86, 90, 91, 93, 94, 95, 96, 98, 100, 101, 102, 104, 105, 106, 116, 118, 119, 120, 121, 122, 123, 124, 125, 126, 127, 132, 133, 134, 137, 138, 140, 141, 142, 143, 144, 146, 147, 148, 149, 151, 152, 153, 154, 155, 157, 159, 161, 162, 164, 165, 166, 168]. In the full‐text analysis, 39 studies [8, 9, 13, 14, 15, 16, 29, 36, 44, 46, 56, 58, 62, 64, 76, 82, 87, 88, 89, 92, 97, 99, 103, 108, 109, 111, 113, 114, 115, 117, 130, 131, 135, 136, 139, 150, 158, 163, 167] were excluded for the following reasons: 18 studies [8, 13, 14, 16, 36, 44, 56, 58, 62, 64, 99, 108, 111, 113, 114, 115, 130, 167] were excluded because they did not report the outcome of interest, 10 studies [29, 76, 82, 87, 88, 89, 97, 103, 150, 158] were excluded because they reported data from a participant cohort that was already included in our analysis from other primary studies, 8 studies [9, 46, 92, 109, 131, 135, 136, 139] were excluded because they investigated unilateral knee arthroplasty, and 3 studies [15, 117, 163] were excluded because it was not possible to extract clear data distraction force.

### Characteristics of the studies included

Of the 116 studies included [1, 2, 3, 4, 5, 6, 7, 10, 11, 12, 17, 18, 19, 20, 21, 22, 23, 25, 26, 27, 28, 30, 31, 33, 34, 35, 37, 38, 39, 40, 41, 42, 43, 45, 49, 55, 57, 59, 61, 63, 65, 66, 67, 68, 69, 70, 71, 72, 73, 74, 75, 77, 78, 79, 80, 81, 83, 84, 85, 86, 90, 91, 93, 94, 95, 96, 98, 100, 101, 102, 104, 105, 106, 116, 118, 119, 120, 121, 122, 123, 124, 125, 126, 127, 132, 133, 134, 137, 138, 140, 141, 142, 143, 144, 146, 147, 148, 149, 151, 152, 153, 154, 155, 157, 159, 161, 162, 164, 165, 166, 168], 9 were RCTs [3, 33, 43, 50, 55, 57, 69, 77, 95] and 107 were non‐RCTs [1, 2, 4, 5, 6, 7, 10, 11, 12, 17, 18, 19, 20, 21, 22, 23, 25, 26, 27, 28, 30, 31, 34, 35, 37, 38, 39, 40, 41, 42, 45, 49, 51, 54, 59, 61, 63, 65, 66, 67, 68, 70, 71, 72, 73, 74, 75, 78, 79, 80, 81, 83, 84, 85, 86, 90, 91, 93, 94, 96, 98, 100, 101, 102, 104, 105, 106, 116, 118, 119, 120, 121, 122, 123, 124, 125, 126, 127, 132, 133, 134, 137, 138, 140, 141, 142, 143, 144, 146, 147, 148, 149, 151, 152, 153, 154, 155, 157, 159, 161, 162, 164, 165, 166, 168]. Of the 107 non‐RCTs, 30 were experimental studies [1, 5, 6, 10, 17, 19, 21, 23, 40, 42, 51, 52, 53, 54, 63, 66, 78, 83, 93, 96, 116, 119, 124, 137, 146, 147, 148, 153, 164, 168], 36 were retrospective case series [2, 34, 37, 38, 39, 49, 59, 68, 70, 73, 74, 75, 79, 81, 84, 86, 90, 91, 94, 98, 105, 118, 120, 121, 123, 132, 134, 143, 144, 149, 154, 155, 157, 159, 166], 12 were retrospective cohort studies [20, 30, 35, 41, 71, 72, 100, 104, 106, 133, 140, 142], 12 were prospective case series [4, 7, 11, 25, 26, 45, 61, 80, 125, 141, 152, 161], 11 were prospective cohort studies [27, 28, 31, 65, 122, 126, 127, 138, 151, 162, 165] and 1 therapeutic study [67]. They were published between 2004 and 2024, and almost all were in English [1, 2, 3, 4, 5, 6, 7, 10, 11, 12, 17, 18, 19, 20, 21, 22, 23, 25, 26, 27, 28, 30, 31, 33, 34, 35, 37, 38, 39, 40, 41, 42, 43, 45, 49, 50, 51, 52, 53, 54, 55, 57, 59, 61, 63, 65, 66, 67, 68, 69, 70, 71, 72, 73, 74, 75, 77, 78, 79, 80, 81, 83, 84, 85, 86, 90, 91, 93, 94, 95, 96, 98, 100, 101, 102, 104, 105, 106, 116, 118, 119, 120, 121, 122, 123, 124, 125, 126, 127, 132, 133, 134, 137, 138, 140, 141, 142, 143, 144, 146, 147, 148, 149, 151, 152, 153, 154, 155, 157, 159, 161, 162, 164, 165, 168], with only one in Chinese [166]. Of the 116 studies included [1, 2, 3, 4, 5, 6, 7, 10, 11, 12, 17, 18, 19, 20, 21, 22, 23, 25, 28, 30, 31, 33, 34, 35, 37, 38, 39, 40, 41, 42, 43, 45, 49, 50, 51, 52, 53, 54, 55, 57, 59, 61, 63, 65, 66, 67, 68, 69, 70, 71, 72, 73, 74, 75, 77, 78, 79, 80, 81, 83, 84, 85, 86, 90, 91, 93, 94, 95, 96, 98, 100, 101, 102, 104, 105, 106, 116, 118, 119, 120, 121, 122, 123, 124, 125, 126, 127, 132, 133, 134, 137, 138, 140, 141, 142, 143, 144, 146, 147, 148, 149, 151, 152, 153, 154, 155, 157, 159, 161, 162, 164, 165, 166, 168], 53 (45.7%) were from Japan [2, 12, 20, 21, 22, 25, 33, 34, 35, 37, 38, 45, 59, 65, 66, 67, 68, 69, 70, 71, 72, 73, 74, 75, 80, 81, 84, 85, 86, 90, 91, 94, 95, 98, 100, 101, 102, 105, 106, 118, 132, 133, 134, 137, 140, 141, 142, 143, 144, 148, 149, 154, 159], 13 (11.2%) from the United States [18, 19, 51, 79, 83, 104, 116, 123, 124, 151, 153, 155, 164], 12 (10.3%) from South Korea [50, 51, 52, 54, 55, 57, 121, 122, 125, 126, 161, 162], 7 (6.0%) from Australia [3, 40, 43, 53, 93, 104, 119], 6 (5.2%) from the Netherlands [5, 26, 27, 28, 30, 31], 4 (3.4%) from China [41, 138, 165, 166] and other countries. Of the 116 studies included [1, 2, 3, 4, 5, 6, 7, 10, 11, 12, 17, 18, 19, 20, 21, 22, 23, 25, 26, 27, 28, 30, 31, 33, 34, 35, 37, 38, 39, 40, 41, 42, 43, 45, 49, 50, 51, 52, 53, 54, 55, 57, 59, 61, 63, 65, 66, 67, 68, 69, 70, 71, 72, 73, 74, 75, 77, 78, 79, 80, 81, 83, 84, 85, 86, 90, 91, 93, 94, 95, 96, 98, 100, 101, 102, 104, 105, 106, 116, 118, 119, 120, 121, 122, 123, 124, 125, 126, 127, 132, 133, 134, 137, 138, 140, 141, 142, 143, 144, 146, 147, 148, 149, 151, 152, 153, 154, 155, 157, 159, 161, 162, 164, 165, 166, 168], 86 reported distraction force in native knees [2, 3, 4, 7, 11, 12, 18, 20, 22, 25, 26, 27, 28, 30, 31, 33, 34, 35, 37, 38, 39, 41, 43, 45, 49, 50, 54, 55, 57, 59, 61, 65, 67, 68, 69, 70, 71, 72, 73, 74, 75, 77, 79, 80, 81, 84, 85, 86, 90, 91, 94, 95, 98, 100, 101, 102, 104, 105, 106, 118, 120, 121, 122, 123, 125, 126, 127, 132, 133, 134, 138, 140, 141, 142, 143, 144, 149, 151, 152, 154, 155, 157, 159, 161, 162, 165, 166], 25 in cadaver knees [6, 10, 17, 19, 21, 23, 40, 51, 53, 54, 63, 66, 78, 83, 93, 96, 116, 119, 124, 137, 146, 147, 148, 153, 168] and 5 in computer models or artificial knees [1, 5, 42, 52, 164].

### Quality assessment

**Figure 2:**
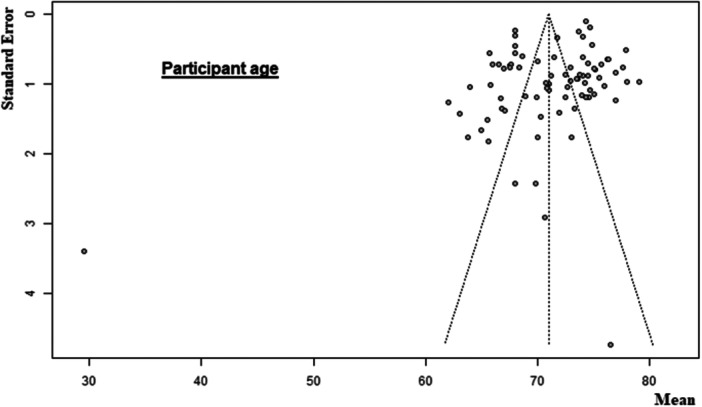
Funnel plot of the participant age.

**Figure 3:**
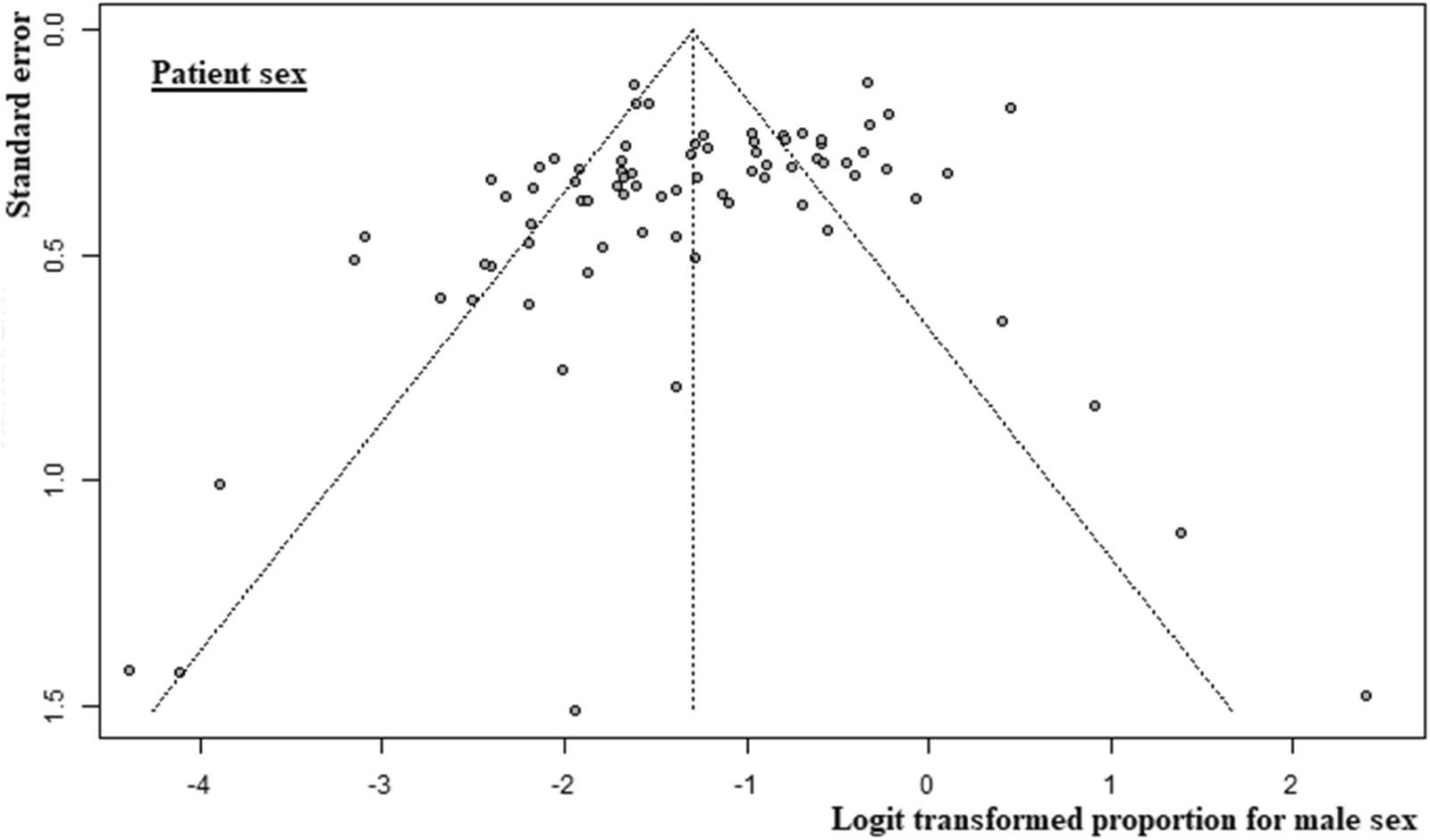
Funnel plot of the participant sex.

**Figure 4:**
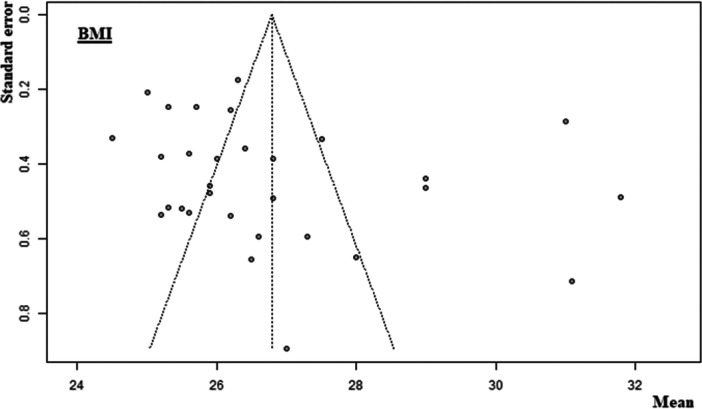
Funnel plot of the BMI. BMI, body mass index.

Of the 107 included non‐RCTs, 65 [1, 5, 7, 10, 11, 18, 20, 23, 25, 26, 28, 30, 31, 34, 35, 37, 40, 42, 49, 52, 53, 54, 61, 63, 65, 72, 78, 80, 81, 83, 85, 86, 90, 91, 94, 96, 98, 100, 101, 104, 105, 106, 116, 118, 120, 121, 123, 124, 125, 126, 127, 132, 134, 137, 138, 140, 144, 149, 152, 154, 159, 161, 162, 164, 168] had a low risk of bias, 35 [4, 6, 12, 17, 19, 21, 22, 27, 38, 39, 41, 45, 51, 67, 68, 70, 71, 73, 74, 75, 93, 102, 119, 122, 133, 141, 142, 143, 146, 147, 148, 151, 157, 165, 166] had a moderate risk of bias, and 7 [2, 59, 66, 79, 84, 153, 155] had a high risk of bias (Table 2). Of the nine included RCTs, six [3, 50, 55, 57, 77, 95] had a low risk of bias, two [43, 69] had a moderate risk of bias, and one [33] had a high risk of bias (Table 3). The funnel plot for participant age (Figure 2) and male sex (Figure 3) showed mild to moderate publication bias, which was confirmed by the Egger test (Egger *p* = 0.0020 and Egger *p* = 0.0044, respectively) (Table 4). Body mass index (BMI) showed no strong evidence of bias (Egger *p* = 0.1862) (Figure 4, Table 4).

**Table 2:** Risk of bias assessment of non‐randomized studies of interventions.

Author	Pre‐intervention		At intervention	Post‐intervention				Overall risk of bias
	Bias due to confounding	Bias in selection of participants into the study	Bias in classification of interventions	Bias due to deviations from intended interventions	Bias due to missing data	Bias in measurement of outcomes	Bias in selection of the reported result	
Amiri et al. [1]	LOW	LOW	LOW	LOW	LOW	LOW	MODERATE	LOW
Asano et al. [2]	MODERATE	MODERATE	MODERATE	LOW	LOW	HIGH	MODERATE	HIGH
Bardou‐Jacquet et al. [4]	MODERATE	LOW	LOW	LOW	LOW	MODERATE	MODERATE	MODERATE
Barink et al. [5]	LOW	LOW	LOW	LOW	LOW	LOW	MODERATE	LOW
Basselot et al. [6]	MODERATE	MODERATE	LOW	LOW	LOW	MODERATE	LOW	MODERATE
Bäthis et al. [7]	MODERATE	LOW	LOW	LOW	LOW	LOW	MODERATE	LOW
Burkhart et al. [10]	MODERATE	LOW	LOW	LOW	LOW	LOW	LOW	LOW
Christen et al. [11]	MODERATE	MODERATE	LOW	LOW	LOW	LOW	LOW	LOW
Christen et al. [12]	MODERATE	MODERATE	LOW	LOW	LOW	LOW	MODERATE	MODERATE
Ferreira et al. [17]	MODERATE	MODERATE	LOW	LOW	LOW	LOW	MODERATE	MODERATE
Fitz et al. [18]	LOW	MODERATE	LOW	LOW	MODERATE	LOW	LOW	LOW
Foge et al. [19]	MODERATE	MODERATE	LOW	LOW	LOW	LOW	MODERATE	MODERATE
Fujimoto et al. [20]	LOW	MODERATE	LOW	LOW	LOW	LOW	LOW	LOW
Gejo et al. [21]	MODERATE	MODERATE	LOW	LOW	LOW	LOW	MODERATE	MODERATE
Gejo et al. [22]	MODERATE	MODERATE	LOW	MODERATE	LOW	MODERATE	LOW	MODERATE
Grifka et al. [23]	MODERATE	LOW	LOW	LOW	LOW	LOW	MODERATE	LOW
Hananouchi et al. [25]	MODERATE	MODERATE	LOW	LOW	LOW	LOW	LOW	LOW
Heesterbeek et al. [26]	LOW	LOW	LOW	LOW	LOW	LOW	MODERATE	LOW
Heesterbeek et al. [27]	MODERATE	MODERATE	LOW	LOW	LOW	LOW	MODERATE	MODERATE
Heesterbeek et al. [28]	LOW	LOW	LOW	LOW	LOW	LOW	MODERATE	LOW
Heesterbeek et al. [30]	MODERATE	LOW	LOW	LOW	LOW	LOW	MODERATE	LOW
Heesterbeek et al. [31]	LOW	LOW	LOW	LOW	LOW	LOW	MODERATE	LOW
Inui et al. [34]	MODERATE	LOW	LOW	LOW	LOW	MODERATE	MODERATE	LOW
Ishida et al. [35]	LOW	MODERATE	LOW	LOW	LOW	LOW	MODERATE	LOW
Itokazu et al. [37]	LOW	LOW	LOW	LOW	LOW	LOW	MODERATE	LOW
Itou et al. [38]	LOW	MODERATE	MODERATE	LOW	LOW	LOW	MODERATE	MODERATE
Jawhar et al. [39]	MODERATE	MODERATE	LOW	LOW	LOW	LOW	MODERATE	MODERATE
Jeffcote et al. [40]	MODERATE	LOW	LOW	LOW	LOW	LOW	MODERATE	LOW
Jia et al. [41]	LOW	MODERATE	LOW	LOW	MODERATE	LOW	MODERATE	MODERATE
Johnston et al. [42]	MODERATE	LOW	LOW	LOW	LOW	LOW	MODERATE	LOW
Kamei et al. [45]	MODERATE	MODERATE	LOW	LOW	LOW	LOW	MODERATE	MODERATE
Keshmiri et al. [49]	MODERATE	LOW	LOW	LOW	LOW	LOW	MODERATE	LOW
Koh et al. [51]	MODERATE	MODERATE	LOW	LOW	LOW	LOW	MODERATE	MODERATE
Koh et al. [52]	LOW	LOW	LOW	LOW	LOW	LOW	MODERATE	LOW
Kuster et al. [53]	MODERATE	LOW	LOW	LOW	LOW	LOW	MODERATE	LOW
Kwak et al. [54]	MODERATE	LOW	LOW	LOW	LOW	LOW	MODERATE	LOW
Ma et al. [59]	HIGH	LOW	LOW	LOW	LOW	LOW	MODERATE	HIGH
Malavolta et al. [61]	MODERATE	LOW	LOW	LOW	LOW	LOW	MODERATE	LOW
Marmignon et al. [63]	LOW	LOW	LOW	LOW	LOW	LOW	LOW	LOW
Matsui et al. [65]	LOW	MODERATE	LOW	LOW	LOW	LOW	MODERATE	LOW
Matsui et al. [66]	HIGH	LOW	LOW	LOW	LOW	LOW	MODERATE	HIGH
Matsumoto et al. [67]	MODERATE	LOW	LOW	LOW	LOW	LOW	MODERATE	MODERATE
Matsumoto et al. [68]	MODERATE	LOW	LOW	LOW	LOW	LOW	MODERATE	MODERATE
Matsumoto et al. [70]	MODERATE	LOW	LOW	LOW	LOW	LOW	MODERATE	MODERATE
Matsumoto et al. [71]	LOW	MODERATE	LOW	LOW	MODERATE	LOW	MODERATE	MODERATE
Matsumoto et al. [72]	LOW	MODERATE	LOW	LOW	LOW	LOW	MODERATE	LOW
Matsumoto et al. [73]	MODERATE	LOW	LOW	LOW	LOW	LOW	MODERATE	MODERATE
Matsumoto et al. [74]	MODERATE	LOW	LOW	LOW	LOW	LOW	MODERATE	MODERATE
Matsuzaki et al. [75]	MODERATE	LOW	LOW	LOW	LOW	LOW	MODERATE	MODERATE
Matziolis et al. [78]	MODERATE	LOW	LOW	LOW	LOW	LOW	MODERATE	LOW
Meere et al. [79]	HIGH	LOW	LOW	LOW	MODERATE	LOW	MODERATE	HIGH
Minoda et al. [80]	LOW	LOW	LOW	LOW	LOW	LOW	MODERATE	LOW
Minoda et al. [81]	MODERATE	LOW	LOW	LOW	LOW	LOW	MODERATE	LOW
Morishige et al. [83]	MODERATE	LOW	LOW	LOW	LOW	LOW	MODERATE	LOW
Moro‐oka et al. [84]	HIGH	LOW	LOW	LOW	LOW	LOW	MODERATE	HIGH
Muratsu et al. [85]	MODERATE	LOW	LOW	LOW	LOW	LOW	MODERATE	LOW
Nagai et al. [86]	LOW	LOW	LOW	LOW	LOW	LOW	MODERATE	LOW
Nakamura et al. [90]	MODERATE	LOW	LOW	LOW	LOW	LOW	MODERATE	LOW
Nakano et al. [91]	MODERATE	LOW	LOW	LOW	LOW	LOW	MODERATE	LOW
Nicholls et al. [93]	MODERATE	MODERATE	LOW	LOW	LOW	LOW	MODERATE	MODERATE
Niki et al. [94]	LOW	LOW	LOW	LOW	LOW	LOW	MODERATE	LOW
Nowakowski et al. [96]	LOW	LOW	LOW	LOW	LOW	LOW	MODERATE	LOW
Ogawa et al. [98]	LOW	LOW	LOW	LOW	LOW	LOW	MODERATE	LOW
Okamoto et al. [100]	LOW	MODERATE	LOW	LOW	LOW	LOW	MODERATE	LOW
Okamoto et al. [101]	MODERATE	LOW	LOW	LOW	LOW	LOW	MODERATE	LOW
Okamoto et al. [102]	MODERATE	MODERATE	MODERATE	LOW	LOW	LOW	MODERATE	MODERATE
Orsi et al. [104]	LOW	MODERATE	LOW	LOW	LOW	LOW	MODERATE	LOW
Oshima et al. [105]	MODERATE	LOW	LOW	LOW	LOW	LOW	MODERATE	LOW
Oshima et al. [106]	LOW	LOW	LOW	LOW	LOW	LOW	MODERATE	LOW
Roth et al. [116]	MODERATE	LOW	LOW	LOW	LOW	LOW	MODERATE	LOW
Sasaki et al. [118]	MODERATE	LOW	LOW	LOW	LOW	LOW	MODERATE	LOW
Schirm et al. [119]	MODERATE	MODERATE	LOW	LOW	LOW	LOW	MODERATE	MODERATE
Seito et al. [120]	LOW	LOW	LOW	LOW	LOW	LOW	MODERATE	LOW
Seo et al. [121]	LOW	LOW	LOW	LOW	LOW	LOW	MODERATE	LOW
Seon et al. [122]	MODERATE	MODERATE	LOW	LOW	LOW	LOW	MODERATE	MODERATE
Shalhoub et al. [123]	MODERATE	LOW	LOW	LOW	LOW	LOW	MODERATE	LOW
Shalhoub et al. [124]	MODERATE	LOW	LOW	LOW	LOW	LOW	MODERATE	LOW
Shin et al. [125]	LOW	MODERATE	LOW	LOW	LOW	LOW	MODERATE	LOW
Song et al. [126]	LOW	MODERATE	LOW	LOW	LOW	LOW	MODERATE	LOW
Sriphirom et al. [127]	MODERATE	LOW	LOW	LOW	LOW	LOW	MODERATE	LOW
Takagi et al. [132]	MODERATE	LOW	LOW	LOW	LOW	LOW	MODERATE	LOW
Takahashi et al. [133]	MODERATE	MODERATE	LOW	LOW	LOW	MODERATE	MODERATE	MODERATE
Takashima et al. [134]	LOW	MODERATE	LOW	LOW	LOW	LOW	MODERATE	LOW
Tanaka et al. [137]	MODERATE	LOW	LOW	LOW	LOW	LOW	MODERATE	LOW
Tang et al. [138]	LOW	MODERATE	LOW	LOW	LOW	LOW	MODERATE	LOW
Terashima et al. [140]	LOW	MODERATE	LOW	LOW	LOW	LOW	LOW	LOW
Tsuboska et al. [141]	LOW	MODERATE	LOW	LOW	LOW	MODERATE	MODERATE	MODERATE
Tsuboska et al. [142]	LOW	MODERATE	LOW	LOW	LOW	MODERATE	MODERATE	MODERATE
Tsukada et al. [143]	LOW	MODERATE	MODERATE	LOW	LOW	MODERATE	LOW	MODERATE
Tsukada et al. [144]	LOW	LOW	MODERATE	LOW	LOW	LOW	LOW	LOW
Viskontas et al. [146]	MODERATE	MODERATE	LOW	LOW	LOW	LOW	MODERATE	MODERATE
Völlner et al. [147]	MODERATE	MODERATE	LOW	LOW	LOW	LOW	MODERATE	MODERATE
Wada et al. [148]	MODERATE	MODERATE	LOW	LOW	LOW	LOW	MODERATE	MODERATE
Wakama et al. [149]	LOW	LOW	MODERATE	LOW	LOW	LOW	MODERATE	LOW
Wakelin et al. [151]	LOW	MODERATE	LOW	LOW	LOW	MODERATE	MODERATE	MODERATE
Walde et al. [152]	MODERATE	LOW	LOW	LOW	LOW	LOW	MODERATE	LOW
Walker et al. [153]	HIGH	MODERATE	LOW	LOW	LOW	LOW	MODERATE	HIGH
Watanabe et al. [154]	LOW	LOW	MODERATE	LOW	LOW	LOW	MODERATE	LOW
Fan et al. [155]	HIGH	MODERATE	MODERATE	LOW	LOW	LOW	MODERATE	HIGH
Wyss et al. [157]	MODERATE	MODERATE	MODERATE	LOW	MODERATE	MODERATE	MODERATE	MODERATE
Yamagami et al. [159]	LOW	LOW	LOW	LOW	LOW	LOW	MODERATE	LOW
Yoon et al. [161]	MODERATE	LOW	LOW	LOW	LOW	MODERATE	LOW	LOW
Yoon et al. [162]	MODERATE	MODERATE	LOW	LOW	LOW	LOW	LOW	LOW
Zapata et al. [164]	LOW	LOW	LOW	LOW	LOW	LOW	LOW	LOW
Zhao et al. [165]	MODERATE	MODERATE	LOW	MODERATE	LOW	MODERATE	MODERATE	MODERATE
Zhao et al. [166]	MODERATE	MODERATE	LOW	MODERATE	LOW	MODERATE	MODERATE	MODERATE
Zimmermann et al. [168]	LOW	LOW	LOW	LOW	LOW	LOW	LOW	LOW

*Note*: (+): low risk of bias; (?): some concerns; (−): high risk of bias.

**Table 3:** Risk of bias assessment of RCTs.

Author	Bias from arising the randomization process	Bias due to deviation from intended interventions	Bias due to missing outcome data	Bias in measurement of the outcome	Bias in selection of the reported result	Overall risk of bias
Babazadeh et al. [3]	LOW	LOW	LOW	LOW	MODERATE	LOW
Higuchi et al. [33]	HIGH	LOW	MODERATE	HIGH	MODERATE	HIGH
Joseph et al. [43]	MEDIUM	LOW	LOW	LOW	MODERATE	MODERATE
Kim et al. [50]	LOW	LOW	LOW	LOW	MODERATE	LOW
Lee et al. [55]	LOW	LOW	LOW	LOW	MODERATE	LOW
Lee et al. [57]	LOW	LOW	LOW	LOW	MODERATE	LOW
Matsumoto et al. [69]	MODERATE	LOW	MODERATE	MODERATE	MODERATE	MODERATE
Matziolis et al. [77]	LOW	LOW	LOW	LOW	LOW	LOW
Nishizawa et al. [95]	LOW	LOW	LOW	LOW	MODERATE	LOW

*Note*: (+): low risk of bias; (?): some concerns; (−): high risk of bias.

Abbreviations: randomized controlled trial.

**Table 4:** Meta‐analysis results summary.

Participant age										
	Studies	Participants	Mean	Confidence interval	*Τ* ^2^	*I* ^2^	Heterogeneity: *p*	Egger bias	Egger *p*	Difference: *p*
Total	82	6089	71.03	69.78–72.28	31.70	0.97	<0.0001	−2.88	0.0020	0.299
Native knee	78	6066	71.49	70.60–72.39	14.89	0.97	<0.0001			
Cadaver knee	4	23	60.47	26.87–94.07	431.12	0.97	<0.0001			

Male sex										
	Studies	Participants	Mean	Confidence interval	*Τ* ^2^	*I* ^2^	Heterogeneity: *p*	Egger bias	Egger p	Difference: *p*
Total	81	6006	0.21	0.18–0.25	0.76	0.84	<0.0001	−1.83	0.0044	0.0010
Native knee	76	5976	0.21	0.18–0.24	0.58	0.85	<0.0001			
Cadaver knee	5	30	0.66	0.27–0.91	1.29	0.20	0.2846			

BMI										
	Studies	Participants	Mean	Confidence interval	*Τ* ^2^	*I* ^2^	Heterogeneity: *p*	Egger bias	Egger *p*	Difference: *p*
Total	30	3047	26.79	26.09–27.48	3.24	0.96	<0.0001	3.04	0.1862	–
Native knee	30	3047	26.79	26.09–27.48	3.24	0.96	<0.0001			
Cadaver knee	0	0	–	–	–	–	–

Abbreviation: BMI, body mass index.

### Cohort of participants

The 116 included primary studies [1, 2, 3, 4, 5, 6, 7, 10, 11, 12, 17, 18, 19, 20, 21, 22, 23, 25, 26, 27, 28, 30, 31, 33, 34, 35, 37, 38, 39, 40, 41, 42, 43, 45, 49, 50, 51, 52, 53, 54, 55, 57, 59, 61, 63, 65, 66, 67, 68, 69, 70, 71, 72, 73, 74, 75, 77, 78, 79, 80, 81, 83, 84, 85, 86, 90, 91, 93, 94, 95, 96, 98, 100, 101, 102, 104, 105, 106, 116, 118, 119, 120, 121, 122, 123, 124, 125, 126, 127, 132, 133, 134, 137, 138, 140, 141, 142, 143, 144, 146, 147, 148, 149, 151, 152, 153, 154, 155, 157, 159, 161, 162, 164, 165, 166, 168] involved a total of 6869 participants who underwent TKA with distraction force measurement. Of these, 6804 procedures (99.0%) were performed on native knees, 81 procedures (0.9%) were performed on cadaver knees, and 8 procedures (0.1%) were performed on computer models or artificial knees. The mean participant age was 71 years, the mean BMI was 26.8 kg/m² and 22.9% of the participants were male. There was no significant difference in participant age between native and cadaver knee studies (*p* = 0.297). The proportion of men in the cadaver group was significantly higher than in the native group, 66% compared to 21% (*p* = 0.0010). More details on the study participants are shown in Table 1.

### Distraction force

**Figure 5:**
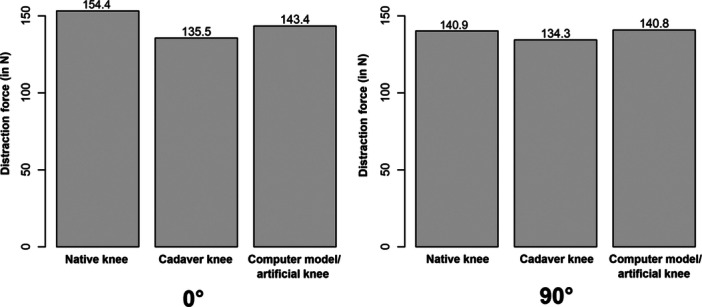
Differences in distraction force applied in TKA between native knee, cadaver knee and computer model/artificial knee. N, Newtons; TKA, total knee arthroplasty.

The mean distraction force in TKA studies was 149.9 ± 45.6 N at extension and 139.5 ± 43.5 N at 90° of knee flexion (Figure 5, Table 5). Using the Kruskal–Wallis test, there were no significant differences in distraction force between native knee, cadaver knee, computer model/artificial knee studies at extension (*p* = 0.2480) and at 90° of knee flexion (*p* = 0.8439) (Table 6). Using the Mann–Whitney *U* test, there were no significant differences between distraction force in TKA in native and cadaver knee studies at 0° of full leg extension (*p *= 0.1130) and at 90° of knee flexion (*p* = 0.6241) (Table 6).

**Table 5:** Means of distraction force applied in TKA at 0° of full leg extension and 90° of knee flexion for native knee, cadaver knee and computer model/artificial knee.

	At 0° of full leg extension		At 90° of knee flexion	
	Studies, *N*	Mean distraction load in ° ±SD (range)	Studies, *N*	Mean distraction load in ° ±SD (range)
Native knee	82	154.4 ± 46.7 (35.0–320.0)	58	140.9 ± 43.4 (14.7–244.7)
Cadaver knee	23	135.5 ± 37.9 (90.0–200.0)	16	134.3 ± 42.0 (80.0–200.0)
Computer model/artificial knee	5	143.4 ± 53.6 (100.0–230.0)	4	140.8 ± 61.5 (100.0–230.0)
Native knee/cadaver knee	105	150.2 ± 45.5 (35.0–320.0)	74	139.5 ± 42.9 (14.7–244.7)
Total	110	149.9 ± 45.6 (35.0–320.0)	78	139.5 ± 43.5 (14.7–244.7)

Abbreviations: N, Newtons; SD, standard deviation; TKA, total knee arthroplasty.

**Table 6:** Differences in distraction force applied in TKA at 0° of full leg extension and 90° of knee flexion, using the Kruskal–Wallis test and the Mann–Whitney *U* test.

Kruskal–Wallis test			
	Kruskal–Wallis $\chi^{2}$	df	*p* **value**
At 0° of full leg extension	2.7888	2	0.2480
At 90° of knee flexion	0.3393	2	0.8439

Mann–Whitney *U* test			
	*W*		*p* **value**
At 0° of full leg extension	1147.0		0.1130
At 90° of knee flexion	501.5		0.6241

Abbreviation: TKA, total knee arthroplasty.

### Sensitivity analysis

The sensitivity analysis, which excluded primary studies with a high risk of bias [2, 33, 59, 66, 79, 84, 153, 155], found no statistically significant differences when compared to the overall set of included studies [1, 2, 3, 4, 5, 6, 7, 10, 11, 12, 17, 18, 19, 20, 21, 22, 23, 25, 26, 27, 28, 30, 31, 33, 34, 35, 37, 38, 39, 40, 41, 42, 43, 45, 49, 50, 51, 52, 53, 54, 55, 57, 59, 61, 63, 65, 66, 67, 68, 69, 70, 71, 72, 73, 74, 75, 77, 78, 79, 80, 81, 83, 84, 85, 86, 90, 91, 93, 94, 95, 96, 98, 100, 101, 102, 104, 105, 106, 116, 118, 119, 120, 121, 122, 123, 124, 125, 126, 127, 132, 133, 134, 137, 138, 140, 141, 142, 143, 144, 146, 147, 148, 149, 151, 152, 153, 154, 155, 157, 159, 161, 162, 164, 165, 166, 168]. The results of the sensitivity analysis are provided in Tables [Link], [Link], [Link] and Figure S1.

## DISCUSSION

This meta‐analysis of 116 primary studies [1, 2, 3, 4, 5, 6, 7, 10, 11, 12, 17, 18, 19, 20, 21, 22, 23, 25, 26, 27, 28, 30, 31, 33, 34, 35, 37, 38, 39, 40, 41, 42, 43, 45, 49, 50, 51, 52, 53, 54, 55, 57, 59, 61, 63, 65, 66, 67, 68, 69, 70, 71, 72, 73, 74, 75, 77, 78, 79, 80, 81, 83, 84, 85, 86, 90, 91, 93, 94, 95, 96, 98, 100, 101, 102, 104, 105, 106, 116, 118, 119, 120, 121, 122, 123, 124, 125, 126, 127, 132, 133, 134, 137, 138, 140, 141, 142, 143, 144, 146, 147, 148, 149, 151, 152, 153, 154, 155, 157, 159, 161, 162, 164, 165, 166, 168] with 6869 participants found that the mean distraction force in TKA studies was 149.9 N at leg extension and 139.5 N at 90° of knee flexion. There were no significant differences in distraction force between native knee, cadaver knee and computer model/artificial knee studies.

These values can be used as benchmarks for intraoperative adjustments, helping to standardize procedures and potentially optimize patient outcomes. The lack of significant differences in distraction force between native knee, cadaver knee and computer model/artificial knee studies further supports the robustness of these findings, suggesting that distraction force in TKA is relatively consistent regardless of the experimental model used. The Kruskal–Wallis test and the Mann–Whitney *U* test found no significant differences in distraction force at both knee extension and 90° of flexion, suggesting that distraction force measurements can be reliably studied in different contexts, including cadaver and computer models. This finding has significant implications for preclinical research, where cadaver and computational models are often used to simulate TKA conditions. The ability to generalize findings from these models to real‐world clinical settings increases their utility, allowing greater flexibility in experimental design and early‐stage research, especially in the absence of live patient data. However, these results should be interpreted with caution. Although no significant differences between models were observed, the relatively small number of cadaver and computer/artificial knee studies (25 and 5, respectively) may limit the generalizability of these findings.

The distraction force is critical to ensure proper tension of the ligaments throughout the range of motion. Inadequate or excessive loading can lead to complications such as joint instability, stiffness, malalignment or early implant wear, ultimately affecting long‐term patient outcomes. It remains unclear whether the extension gap should be measured in full extension where the posterior capsule provides mediolateral stability or in 10° of knee flexion where the posterior capsule is relaxed, and mediolateral stability relies on the collateral ligaments.

Implant design, whether cruciate‐retaining (CR) or posterior‐stabilized (PS), has an impact on both the extension and flexion gap. While the resection of the ACL increases the extension gap by 1.2 mm, sectioning the posterior cruciate ligament additionally increases the gap by 1.3 mm [47, 48].

A standard mean distraction force of 149 N at full extension and 139 N at 90° flexion provides surgeons with important intraoperative values to optimize joint stability and prosthesis function because surgeon's intraoperative assessment has shown high inaccuracy in proper TKA balance [60].

Compression force of 60–136 N has been recommended in the medial compartment using an intraoperative pressure sensor (VERASENSE‐System, OrthoSensor) [160]. In contrast, the developers of this sensor technology have advised a compression force of 240 and 200 N in the medial and lateral compartments, respectively, which is significantly higher than the forces reported in the current meta‐analysis [24]. The release of the deep medial collateral ligament (MCL) and removal of cruciate ligaments and the menisci may necessitate higher forces in both extension and flexion gaps during bony preparation to provide appropriate knee stability and might be the reason for this discrepancy.

The quality assessment of the included studies demonstrated that most of the non‐randomized studies had a low risk of bias (64 out of 105), confirming the reliability of their own results. However, the presence of seven non‐RCTs and one RCT with a high risk of bias indicates that some studies may have methodological limitations that could affect the accuracy of their reported results. Potential sources of bias, such as different techniques for measuring distraction or inconsistent reporting standards, may introduce variability into the data. This underscores the need for future research to adhere to rigorous methodological standards, including standardized protocols for measuring and reporting distraction.

The fact that most studies were conducted in Japan (44.7%) raises an interesting point about the geographical distribution and the potential for regional differences in TKA practice. Although distraction force did not differ significantly between studies from different countries, future research could investigate whether regional differences in surgical technique, patient demographics or healthcare systems might influence intraoperative practice and outcomes. This would help determine whether the reported mean distraction force was universally applicable or needed adjustment for specific populations or healthcare environments.

Publication bias was assessed using funnel plots for key variables such as BMI and male sex distribution. The funnel plot for BMI shows a relatively symmetrical distribution, indicating minimal publication bias (Figure 4). However, there are a few outliers, suggesting the possible presence of unpublished or under‐reported data in studies with higher mean BMIs. The funnel plot for participant age proportions shows a wider range, with some studies falling outside the expected range, suggesting mild to moderate publication bias, which means that studies with certain age distributions, particularly older or younger participants, may be under‐reported. Similarly, the funnel plot for male sex suggests mild to moderate publication bias, particularly in under‐reporting data with low proportions of male participants. The Egger test further confirmed significant bias in participant age (Egger *p* = 0.0020) and male sex (Egger *p* = 0.0044), whereas BMI showed no strong evidence of bias (Egger *p* = 0.1862) (Table 4). These results suggest that although the overall data integrity is sound, certain demographic variables, particularly male sex distribution, may be underrepresented in the included studies.

Most surgeons aim for symmetrical rectangular extension and flexion gaps during surgery, however the lateral femorotibial compartment is more mobile than the medial one, in the natural knee, leading to more laxity on the lateral side. A significant range of stiffness has been reported ranging from 60 to 80 N/mm for the lateral collateral ligament (LCL) and from 34 to 97 N/mm for the MCL [156]. However, few studies described the stiffness transition point as the point where there is no difference in extension (MCL = 52.3 N ± 1.6, LCL = 54.5 N ± 1.7), but in flexion, the LCL (59.3 N ± 2.3) showed a higher stiffness than the MCL (48.3 N ± 2.1) [31]. This raises the question whether an asymmetric force should be applied in the medial and lateral compartments, especially in flexion. A recent study described a robot‐assisted surgical technique that implemented this theory, but the impact on patient's clinical outcomes needs to be studied [145].

## CONCLUSION

This meta‐analysis is the first to quantify distraction force in TKA, providing essential reference values of 149.9 N at 0° extension and 139.5 N at 90° flexion. These findings offer valuable guidelines for intraoperative soft tissue management and joint alignment during TKA procedures. The consistency of distraction force across different experimental models suggests that these values are broadly applicable.

## AUTHOR CONTRIBUTIONS

Nikolai Ramadanov and Roland Becker performed the literature analysis and wrote the manuscript. Maximilan Voss, Robert Hable and Nikolai Ramadanov performed the statistics. Mahmut Enes Kayaalp, Jonathan Lettner, Pier Indelli and Reha Tandogan revised the manuscript.

## CONFLICT OF INTEREST STATEMENT

Roland Becker is a board member of ESSKA and an editorial board member of KSSTA and received a speaker honorarium from ENOVIS and Rimasys. Reha Tandogan is the associated editor of KSSTA and receives Consulting fees from Stryker, Smith&Nephew, Menarini Pharma and Santa Farma Pharma. Mahmut Enes Kayaalp is the associated editor of KSSTA. The remaining authors declare no conflicts of interest.

## ETHICS STATEMENT

The ethics statement is not available.

## CLINICAL TRIAL REGISTRATION

Prospero: CRD42023434713.

## Supporting information

Supporting information.

Supporting information.

Supporting information.

Supporting information.

Supporting information.

## Data Availability

Data from other studies were solely used.
