# Supplementary figures and images for: Mean distraction force applied in tension‐controlled ligament‐balanced total knee arthroplasty: A systematic review and meta‐analysis
Source: Knee Surg Sports Traumatol Arthrosc. 2025 Feb 26;33(7):2498–526. doi: 10.1002/ksa.12629 (PMC12205427; doi:10.1002/ksa.12629)

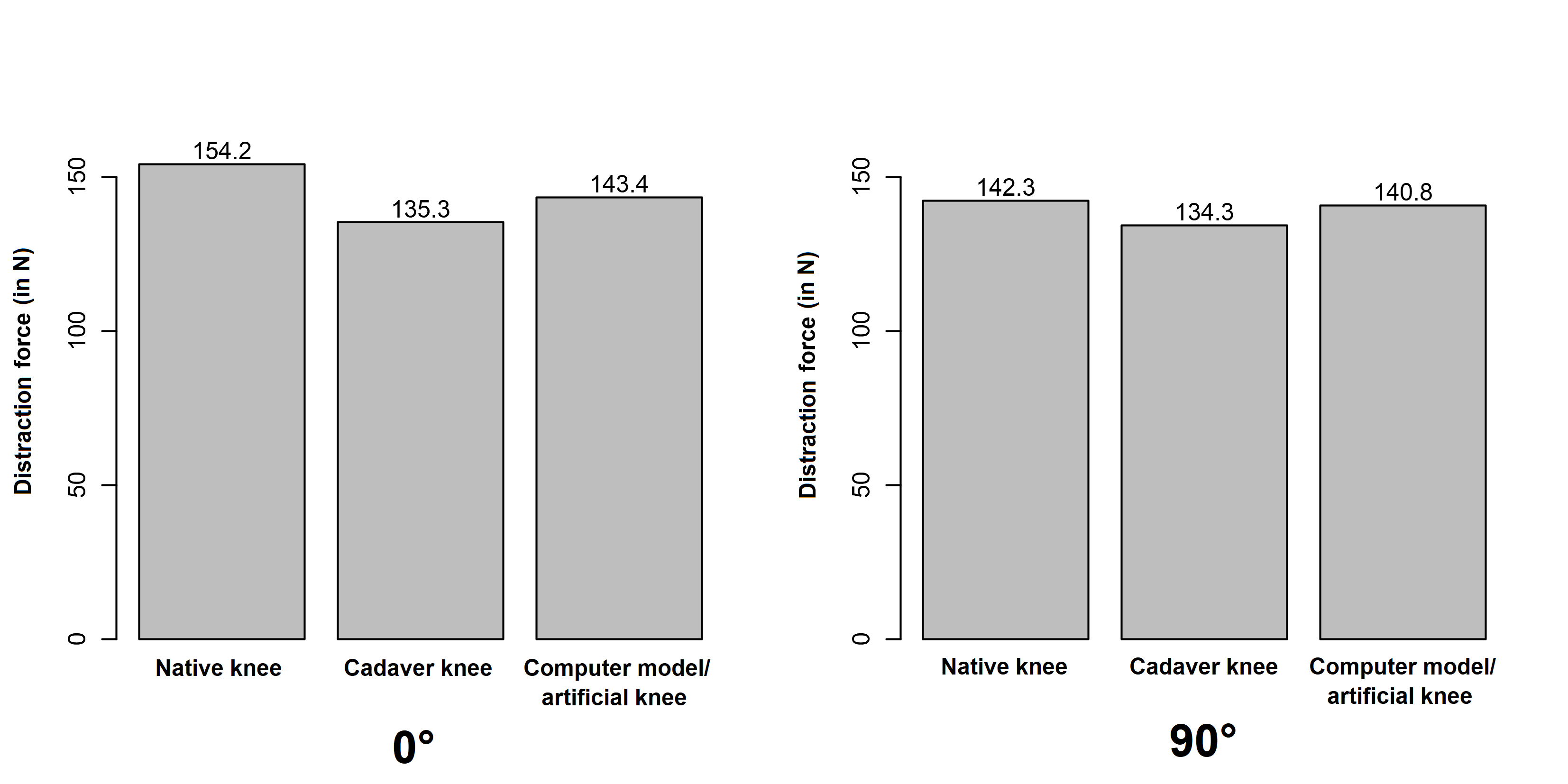

Supplement: Supplementary file 1 — Supporting information. [file KSA-33-2498-s004.png]
