# Supplementary material for: Mean distraction force applied in tension‐controlled ligament‐balanced total knee arthroplasty: A systematic review and meta‐analysis
Source: Knee Surg Sports Traumatol Arthrosc. 2025 Feb 26;33(7):2498–526. doi: 10.1002/ksa.12629 (PMC12205427; doi:10.1002/ksa.12629)
Supplement: Supplementary file 2 — Supporting information. [file KSA-33-2498-s001.docx]

| **Participant age** | | | | | | | | | | |
| --- | --- | --- | --- | --- | --- | --- | --- | --- | --- | --- |
|  | Studies | Participants | Mean | Confidence interval | tau^2^ | I^2^ | Heterogenity: p | Egger bias | Egger p | Difference: p |
| Total | 81 | 6012 | 71.055 | 69.79 to 72.32 | 32.09 | 0.97 | < 0.0001 *** | -2.86 | 0.0025 ** | 0.296 |
| Native knee | 77 | 5989 | 71.53 | 70.62 to 72.43 | 15.01 | 0.97 | < 0.0001 *** |  |  |  |
| Cadaver knee | 4 | 23 | 60.47 | 26.87 to 94.07 | 431.12 | 0.97 | < 0.0001 *** |  |  |  |
| **Male sex** | | | | | | | | | | |
|  | Studies | Participants | Mean | Confidence interval | tau^2^ | I^2^ | Heterogenity: p | Egger bias | Egger p | Difference: p |
| Total | 78 | 5876 | 0.21 | 0.18 to 0.25 | 0.79 | 0.85 | < 0.0001 *** | -1.86 | 0.0054 ** | 0.0001 *** |
| Native knee | 74 | 5849 | 0.21 | 0.18 to 0.24 | 0.60 | 0.85 | < 0.0001 *** |  |  |  |
| Cadaver knee | 4 | 27 | 0.72 | 0.45 to 0.89 | 0.21 | 0.00 | 0.6142 |  |  |  |
| **BMI** | | | | | | | | | | |
|  | Studies | Participants | Mean | Confidence interval | tau^2^ | I^2^ | Heterogenity: p | Egger bias | Egger p | Difference: p |
| Total | 30 | 3047 | 26.79 | 26.09 to 27.48 | 3.24 | 0.96 | < 0.0001 *** | 3.04 | 0.1862 | --- |
| Native knee | 30 | 3047 | 26.79 | 26.09 to 27.48 | 3.24 | 0.96 | < 0.0001 *** |  |  |  |
| Cadaver knee | 0 | 0 | --- | --- | --- | --- | --- |  |  |  |
