# Supplementary material for: Mean distraction force applied in tension‐controlled ligament‐balanced total knee arthroplasty: A systematic review and meta‐analysis
Source: Knee Surg Sports Traumatol Arthrosc. 2025 Feb 26;33(7):2498–526. doi: 10.1002/ksa.12629 (PMC12205427; doi:10.1002/ksa.12629)
Supplement: Supplementary file 3 — Supporting information. [file KSA-33-2498-s002.docx]

|  | **At 0° of full leg extension** | | **At 90° of knee flexion** | |
| --- | --- | --- | --- | --- |
|  | **Studies, N** | **Mean distraction load in ° ±SD (range)** | **Studies, N** | **Mean distraction load in °** ±**SD (range)** |
| **Native knee** | 77 | 154.2 ± 45.9 (35.0 – 320.0) | 55 | 142.3 ± 43.9 (14.7 - 244.7) |
| **Cadaver knee** | 21 | 135.3 ± 39.7 (90.0 – 200.0) | 16 | 134.3 ± 42.0 (80.0 – 200.0) |
| **Computer model/artificial knee** | 5 | 143.4 ± 53.6 (100.0 – 230.0) | 4 | 140.8 ± 61.5 (100.0 – 230.0) |
| **Native knee/cadaver knee** | 98 | 150.2 ± 45.2 (35.0 – 320.0) | 71 | 140.5 ± 43.3 (14.7 - 244.7) |
| **Total** | 103 | 149.8 ± 45.3 (35.0 – 320.0) | 75 | 140.5 ± 43.9 (14.7 - 244.7) |
