# Supplementary material for: Mean distraction force applied in tension‐controlled ligament‐balanced total knee arthroplasty: A systematic review and meta‐analysis
Source: Knee Surg Sports Traumatol Arthrosc. 2025 Feb 26;33(7):2498–526. doi: 10.1002/ksa.12629 (PMC12205427; doi:10.1002/ksa.12629)
Supplement: Supplementary file 4 — Supporting information. [file KSA-33-2498-s003.docx]

| **Kruskal-Wallis test** | | | |
| --- | --- | --- | --- |
|  | **Kruskal-Wallis** $\boldsymbol{\chi}^{\boldsymbol{2}}$ | **df** | **p value** |
| **At 0° of full leg extension** | 2.5042 | 2 | 0.2859 |
| **At 90° of knee flexion** | 0.5003 | 2 | 0.7787 |
