# Supplementary material for: Mean distraction force applied in tension‐controlled ligament‐balanced total knee arthroplasty: A systematic review and meta‐analysis
Source: Knee Surg Sports Traumatol Arthrosc. 2025 Feb 26;33(7):2498–526. doi: 10.1002/ksa.12629 (PMC12205427; doi:10.1002/ksa.12629)
Supplement: Supplementary file 5 — Supporting information. [file KSA-33-2498-s005.docx]

SUPPLEMENTAL TABLES

Supplemental Table 1: Meta-analysis results summary. BMI: Body Mass Index;

Supplemental Table 2: Means of distraction load force applied in TKA at 0° of full leg extension and 90° of knee flexion for native knee, cadaver knee and computer model/artificial knee. Sensitivity analysis excluding studies with a high risk of bias. TKA: total knee arthroplasty; N: Newtons; SD: standard deviation;

Supplemental Table 3: Differences in distraction load force applied in TKA at 0° of full leg extension and 90° of knee flexion, using the Kruskal-Wallis test and the Mann-Whitney U test. Sensitivity analysis excluding studies with a high risk of bias. TKA: total knee arthroplasty;

SUPPLEMENTAL FIGURES

Supplemental Figure 1: Differences in distraction force applied in TKA between native knee, cadaver knee and computer model/artificial knee. Sensitivity analysis excluding studies with a high risk of bias. *TKA: total knee arthroplasty; N: Newtons;*
